# First Phytochemical Profiling and *In-Vitro* Antiprotozoal Activity of Essential Oil and Extract of *Plagiochila porelloides*

**DOI:** 10.3390/molecules28020616

**Published:** 2023-01-07

**Authors:** Anaïs Pannequin, Joëlle Quetin-Leclercq, Jean Costa, Aura Tintaru, Alain Muselli

**Affiliations:** 1 Université de Corse, UMR CNRS 6134 Sciences Pour l’Environnement, Laboratoire Chimie des Produits Naturels, BP 52, 20250 Corte, France; 2 UCLouvain, Louvain Drug Research Institute, Pharmacognosy Group, Avenue E. Mounier 72, B-1200 Brussels, Belgium; 3 Aix Marseille Univ, CNRS, Centre Interdisciplinaire de Nanoscience de Marseille, UMR7325, 13288 Marseille, France

**Keywords:** bryophytes, *plagiochila porelloides*, humbertiane, farnesane, sesquiterpenes, antiprotozoal

## Abstract

Volatiles metabolites from the liverwort *Plagiochila porelloides* harvested in Corsica were investigated by chromatographic and spectroscopic methods. In addition to already reported constituents, three new compounds were isolated by preparative chromatography and their structures were elucidated by mass spectrometry (MS) and NMR experiments. Hence, an atypic aliphatic compound, named 1,2-dihydro-4,5-dehydronerolidol and two isomers, (E) and (Z), possessing an unusual humbertiane skeleton (called *p*-menth-1-en-3-[2-methylbut-1-enyl]-8-ol) are newly reported and fully characterized in this work. The in vitro antiprotozoal activity of essential oil and extract of *P. porelloides* against *Trypanosoma brucei brucei* and *Leishmania mexicana mexicana* and cytotoxicity were determined. Essential oil and Et_2_O extract showed a moderate activity against *T. brucei* with IC_50_ values: 2.03 and 5.18 μg/mL, respectively. It is noteworthy that only the essential oil showed a high selectivity (SI = 11.7). Diethyl oxide extract exhibited moderate anticancer (cancerous macrophage-like murine cells) activity and also cytotoxicity (human normal fibroblast) with IC_50_ values: 1.25 and 2.96 μg/mL, respectively.

## 1. Introduction

Bryophytes are largely located in various ecosystems characterized by humid climates, such as lakes, rivers, swings, etc., where the water, an essential element for their development and sexual reproduction, is abundantly present. Considered as the oldest green plants, they are the first vegetables that adapted to terrestrial life 500 million years ago [1]. Furthermore, following their taxonomy, the bryophytes are classified among pteridophytes and algae, and could by sub-divided into three coordinate phyla: liverworts (Marchantiophyta or Hepaticae), mosses (Bryophyta) and hornworts (Anthocerotophyta). Nowadays, approximatively 25,000 species of bryophytes were identified and disseminated worldwide. Among those, 1800 species are located in Europe and almost 75% were identified in French territory [1].

In particular, Corsica has more than 500 species of bryophytes spread over all the vegetation levels of the island [2]. Most of the bryophytes live in fresh and humid places but they are also found in dry and open habitats. They are also present in running water, around streams and lakes, in marshes and bogs. In addition, these plants that contribute significantly to flora diversity and play an essential role in the functioning of many ecosystems (peat bogs, forests, etc.). Bryophytes constitute an important plant biomass available throughout the year, which is not yet economically exploited. To our knowledge, the bryophytes of Corsica have been the subject of only one phytochemical study carried out earlier by our group [3,4]. Nevertheless, even if bryophytes are currently poorly investigated, based on the results reported by the few studies conducted on this topic, they are depicted as “the pharmacy of tomorrow” [5,6,7,8,9]. For all these reasons, the characterization of the molecular constituents of essential oils, volatile fractions, and extracts produced from the bryophytes, combined with their screening of active compounds, constitute an interesting scientific challenge to be performed at the scale of the Corsican region.

*Plagiochila* species is the largest genus in the Marchantiophyta, with at least 1600 varieties [10]. This genus is characterized not only by morphological diversity but also by a huge chemical variety of volatile and non-volatile compounds. From the composition of solvent extracts of almost sixty species of *Plagiochila*, and based on their skeleton, the author distinguished twelve types [10] as follows I: 2,3-secoaromadendrane sesquiterpene-type (subdivided depending on the degree of oxidation and acetylation of 2,3-secoaromadendrane); II: bibenzyl-type; III: cuparane-herbertane sesquiterpene-type; IV: bibenzyl-cuparane-herbertane-type; V: gymnomitrane (barbatane)-bicyclogermacrane sesquiterpene-type; VI: bicyclogermacrane-spathulenol sesquiterpene-type; VII: pinguisane sesquiterpene-type; VIII: 2,3-secoaromadendrane-sesquiterpene lactone-type; IX: cyclic bis-bibenzyl-2,3-secoaromadendrane-type; X: sesquiterpene lactone-type; XI: epiverrucosane type and XII: fusicoccane-labdane type. According to two studies concerning compounds extracted by diethyl oxide, *P. porelloides* can be integrated to type I. The first report of *P. porelloides* compounds led to the isolation 3α-acetoxybicyclogermacrene, bicyclogermacrene, plagiochilines A, -C, -D et -H as the main constituents from the Swiss species [11]. The second investigation reported on the isolation of three sesquiterpene esters from a German sample, derived from mono esterification of a 2,3-secoaromadendrane-type sesquiterpenoid by different fatty acids, in addition to spathulenol [12].

The volatiles constituents of the *Plagiochila* genus have been poorly studied. To our knowledge, only four studies have been carried out on the chemical composition of the essential oils prepared from six *Plagiochila* species: *P. biffaria* [13,14], *P. maderensis*, *P. retrorsa*, *P. stricta* [13], *P. asplenioides* [15] and *P. ovalifolia* [16] (see Table 1). The bibliographic data highlighted a chemical variability according to each studied species. However, the richness in hydrocarbon monoterpenes and oxygenated sesquiterpenes is the common point of all reported varieties. Moreover, all the investigated species constituted a source of new compounds. In *P. bifaria*, three eudesmane type sesquiterpenes, eudesm-4-en-6-one, eudesm-4(15)-en-6-one, 7-hydroxyeudesm-4-en-6-one were isolated and identified as new natural products [14]. In *P. asplenioides*, one aromadendrane sesquiterpene, aromadendra-1(10),3-diene, two aromatic sesquiterpene hydrocarbons, bisabola-1,3,5,7(14)-tetraene and bisabola-1,3,5,7-tetraene, three sesquiterpene oxides, muurolan-4,7-peroxide, plagiochilines W and X were described for the first time [15].

The aim of the present work was to investigate the volatile metabolites of *P. porelloides* prepared by hydrodistillation (essential oil and hydrosol), hexane and diethyl oxide cold-extractions and microwave-assisted extractions using a combination of techniques involving liquid Column Chromatography, GC/FID (using retention indices), GC-MS (EI), HRMS and NMR spectroscopy (^1^H-, ^13^C- and 2D-NMR). As liverwort chemicals are generally very complex mixtures; the identification of components depends on the existing database records and therefore, an important part of our study is dedicated to the identification of components not recorded in MS-libraries [17]. We are reporting here the isolation and structure determination of three unknown natural products showing farnesane and humbertiane skeletons, respectively.

Finally, the cytotoxicity and some antiprotozoal activities of the bulk extracts were investigated in vitro, to evaluate their potential pharmacological properties. Generally, for in vitro screening phase, a molecule is considered to have strong antiparasitic activity if its median inhibitory concentration (IC50) is below 1 µg·mL^−1^. For complex mixtures such as essential oils, the activity becomes interesting when its IC50 is less than 12.5 µg·mL^−1^. When the selectivity index (SI) is greater than five, then the sample is considered a hit and is therefore likely to proceed to the in vivo stage [18]. Neglected tropical diseases (NTDs) are a group of communicable diseases that prevail in tropical and subtropical conditions in about 150 countries and affect more than one billion people, mainly in the world’s poorest people, and are especially common in tropical areas. They include Human African Trypanosomiasis (or African Sleeping Sickness, HAT) and Leishmaniasis caused by *Trypanosoma brucei* (*T.b*) and some twenty species of *Leishmania,* respectively [19]; some forms are lethal for humans. Another common characteristic of these diseases is the absence of an efficient treatment which would not cause toxicity, resistance or other side-effects. Several essential oils are known to possess antimicrobial properties and could also be considered as a source of new antiparasitic compounds [20].

## 2. Results and Discussion

To carry out a more exhaustive study of the volatile metabolites of *P. porelloides*, samples were prepared by four different extraction procedures, starting each time from new dried plant material. In this context, essential oil and hydrosol were obtained by hydrodistillation, solvent extracts by cold maceration and assisted microware extractions as well as the volatiles were sampled using SPME. The identification of components involved a methodology first based on the comparison of RI and MS data with those contained in the in-house library or commercial libraries. After this preliminary analysis, components matched by standards from the in-house library were considered as definitely identified while components matched only by commercial library database needed identification-confirmation. In the present work, several components remained unidentified. So, preparative liquid chromatography and additional NMR experiments were carried out to achieve an unambiguous compound identification, as well as the complete NMR assignment.

Our study allowed for the identification of 58 compounds representing 76.9% of the essential oil (EO) and 52.6% of the hydrosol extract (HY), 82.6% and 77.9% of hexane and diethyl oxide solvent extracts (EXT_H_ and EXT_O_), 89.4% of microwave extract (MW) and 90.3% volatile fraction (VF). Among them, the presence of three unknown sesquiterpenoids was revealed in the diethyl oxide extract and the essential oil of *P. porelloides*.

### 2.1. Resolution of Ambiguous Identifications of Sesquiterpenes

**Figure 1:**
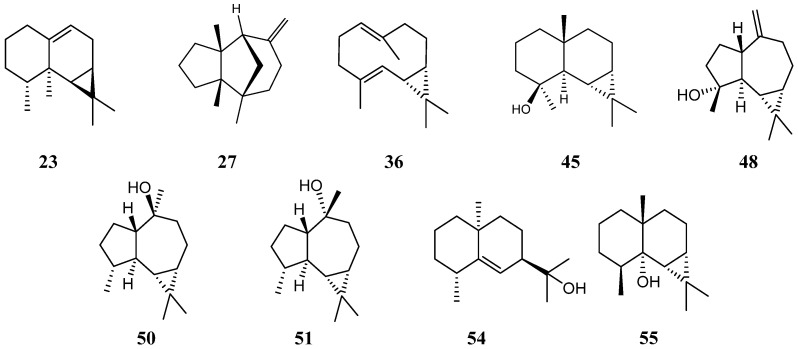
Structures of *P. porelloides* essential oil components identified by GC-MS and ensured by NMR.

Preparative liquid chromatography of *P. polleroides* essential oil was performed to obtained rich-sesquiterpene fractions. As GC-MS identification of sesquiterpenes in complex mixture can be a complex task [21], unambiguous identification of components **45**, **48**, **50**, **51, 54** and **55** were definitively established using NMR-Extraction procedure [22]. Among these, the presence of the isomers spathulenol **48**, globulol **50** and viridiflorol **51** with close RI and mass spectra was confirmed by comparison of their ^13^C-NMR data with those described in the literature. The same procedure allowed the identification of 4-epi-maaliol **45**, rosifoliol **54**, maalian-5-ol **55** (Figure 1).

The compounds **23**, **27** and **36** were concentrated in the hydrocarbon fraction of the essential oil and identified as aristolene **23**, β-barbatene **27** and bicyclogermacrene **36** (Figure 1).

### 2.2. Structural Elucidations of New Natural Compounds

**Figure 2:**
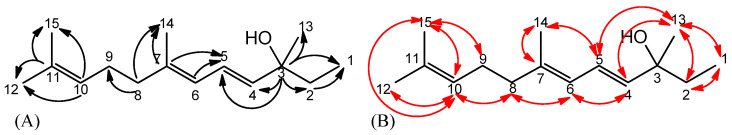
Long range ^13^C-^1^H HMBC (**A**) and NOE (**B**) correlations of **58.**

**Figure 3:**
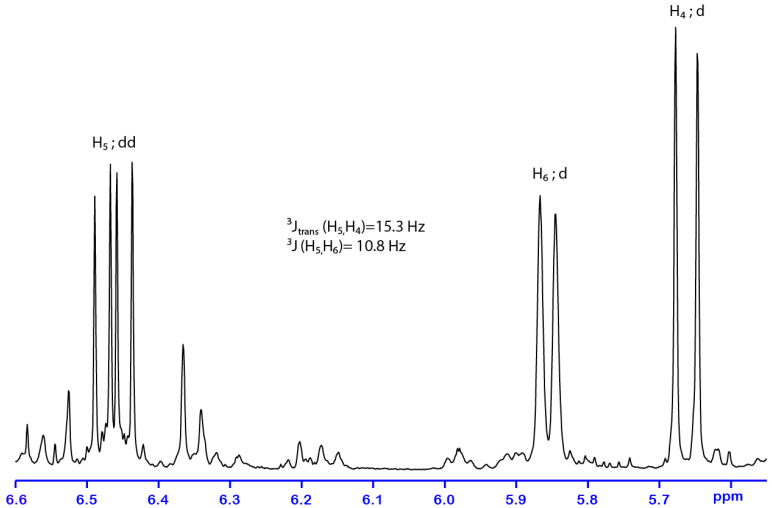
H-H coupling pattern of the ethylenic system of **58**.

Column chromatography of *P. porelloides* EXT_O_ was carried out using a gradient of polarity with hexane and diisopropyl oxide, which produced two fractions in which **58** (65%) were isolated from the polar fraction. EI mass spectra of **58** exhibited a base peak at *m*/*z* 107 and a signal at *m*/*z* 222, which could be attributed to the molecular ion. ESI (+)-HRMS measurements confirms the molecular formula C_15_H_26_O, (detected ion C_15_H_26_ONa^+^ (*m*/*z*)_exp_ 245.1879 and (*m*/*z*)_th_ 245.1876, error +1.2 ppm). The formula indicated a saturation degree of 3. The ^1^H-NMR (CDCl_3_, 300 K) spectrum of **58** (Table 2, Figure S1) showed the presence of five methyl groups δ 1.3, 1.6, 1.7, 1.8 (s, H_13_, H_15_, H_12_ and H_14_, respectively) and δ 0.9 (t, *J* = 7.44 Hz, H_1_) and four olefinic protons δ 5.1 (t, *J* = 6.0 Hz, H_10_), δ 5.6 (d, *J* =15.3 Hz, H_4_), δ 5.9 (d, *J* = 10.8 Hz, H_6_) and δ 6.5 (dd, *J* = 15.3, 10.8 Hz, H_5_). The ^13^C-NMR spectrum contained 15 resonances (Figure S2): five methyls, three methylenes, four methines and three quaternary carbons. The occurrence of a quaternary carbon at 73.38 ppm suggested the presence of a tertiary alcohol. Spectral data were similar to those of nerolidol, which suggests a farnesane-type compound. The HSQC and HMBC correlations (Figure 2A, Figures S3 and S4) and NOE correlations (Figure 2B and Figure S5) indicated that the oxygenated carbon (C_3_) was correlated with the methyl (C_13_), the methylene group (C_2_) and the methine (C_4_). The signal between 5.5 and 6.6 ppm of ^1^H-NMR spectrum showed coupling of methine protons (H_4_, H_5_ and H_6_) (Figure 3). This correlation indicated the presence of a conjugated ethylenic system. The signal of H_5_ is observed as a doublet of doublets pattern due to its interaction with H_6_ and H_4_; the corresponding ^3^J coupling constant values are 10.8 and 15.3 Hz, respectively. The coupling constant ^3^J_H5,H6_, indicated the E-configuration of the diene fragment [23]. In addition, the coupling constant ^3^J_H4,H5_ (15.6 Hz) corresponds to an E-configuration of the C_4_-C_5_ double bond, confirmed by NOE correlation among H_5_ and H_13_. The configuration of the C_6_-C_7_ double bond was established from NOE correlation between H_6_ → H_8_ and H_5_ → H_14_, respectively, suggesting an E configuration. Finally, the structure of compound **58** was determined as 1,2-dihydro-4,5-dehydronerolidol. 

**Figure 4:**
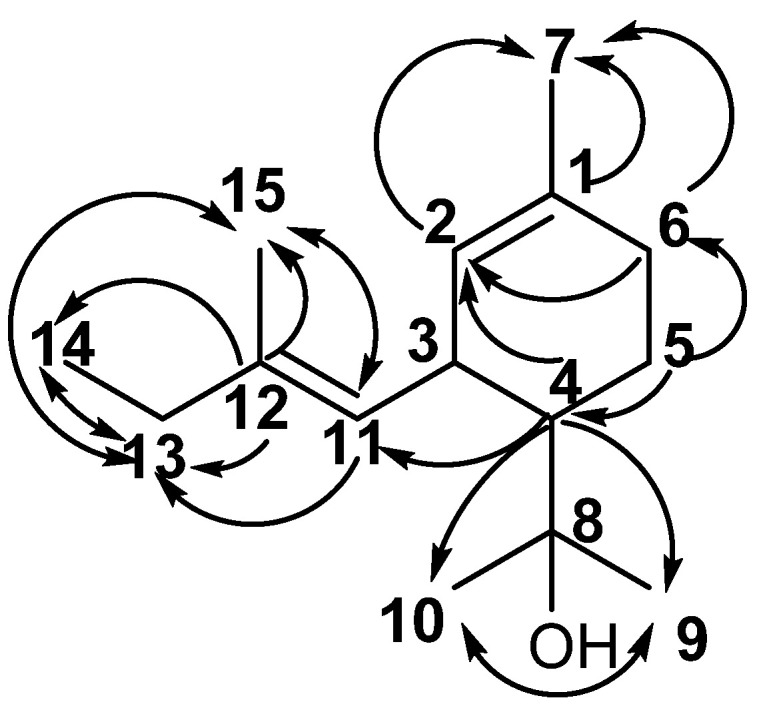
Structure of **42a** and schematic representation of HMBC correlations.

**Figure 5:**
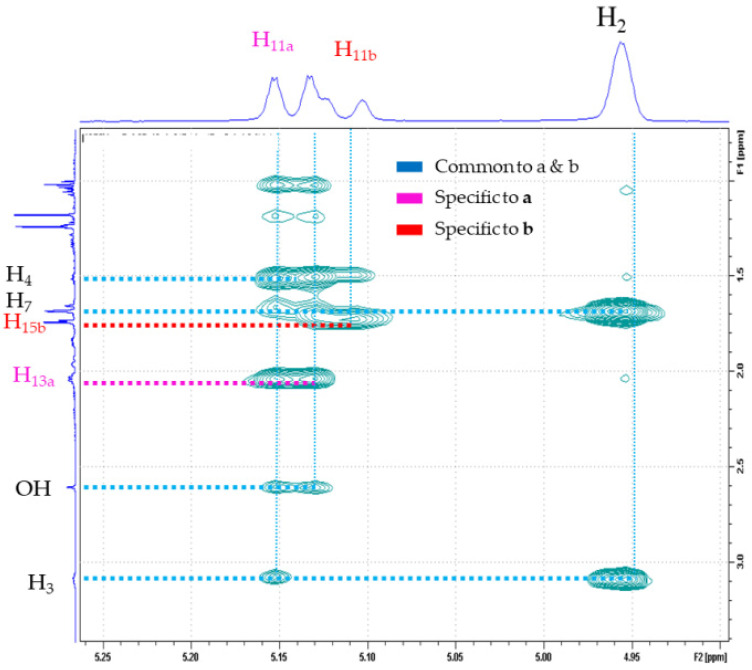
Zoom of NOESY spectrum showing specific correlations used to resolve stereochemistry of the acyclic double bond in **42a,b**.

Compounds **42a** and **42b** were isolated from the fraction **F27** (12 mg) obtained from *P. porelloides* essential oil by column chromatography using hexane and diisopropyl oxide (90:10). Both apolar and polar GC chromatograms of **F27** exhibited one major signal which amounted for 70% of the FID-response. The ^13^C-NMR spectra of **F27** exhibited 29 signals including one carbon atom at δ_c_ 74.4 ppm with double relative intensity. These observations support the hypothesis of the occurrence of a mixture of diastereoisomeres. While the molecular ion of **42** has not been observed, the EI-MS spectra showed a base peak at *m*/*z* 107; the other intense fragment ion, detected at *m*/*z* 59 suggests the presence of a 2-hydroxy isopropyl group, characteristic of tertiary alcohol [24]. ESI (+)-HRMS measurements allowed to determine the molecular formula C_15_H_26_O, (detected ion C_15_H_26_ONa^+^ (*m*/*z*)_exp_ 245.1878 and (*m*/*z*)_th_ 245.1876, error +0.8 ppm). According to the δ_C_ intensities, two sets of resonances with a ratio (2:1) assigned to **42a** and **42b,** respectively, could be extracted from the ^13^C-NMR spectra of **F27** (Figure S7). An Attached Proton Test (APT) experiment confirms the molecular formula C_15_H_26_O for both isomers and the assignment of the 15 carbon signals was carried out as follow: five methyl groups, three methylenes, four methynes of which two ethylenics at δ_C_ 124.88 and 129.59 ppm and three quaternary carbons at δ_C_ 74.38, 137.34 and 134.16 ppm (Table 3). Two-dimensional HSQC, HMBC and COSY experiments (Figures S8 and S9) confirmed the structure of p-menthane framework displaying a side chain composed of five carbon atoms, three of which have sp^3^ hybridization and two of sp^2^ type, forming a double bond (Figure 4). The isopropyl alcohol group was confirmed by HMBC assignment, where appeared the correlations between protons of both methyl terminal groups (δ_H_ at 1.17 and 1.24 ppm, respectively) and the quaternary carbon atom at δ_C_ 74.38 ppm. Moreover, our ^1^H and ^13^C NMR data are in good agreement with those of α-terpineol, an alcohol monoterpene with p-menth-1-en-8-ol structure. Regarding the side chain assignment, the δ_CH2_ and one δ_CH3_ of one isomer exhibited excessive Δδ relative to the other (7.4 ppm and 6.3 ppm, respectively), suggesting steric γ-effects generated by double bond stereochemistry. As ^1^H-coupling constants of the allylic system were not sufficiently to resolve the stereochemistry, NOESY experiments were acquired to elucidate the spatial proximities determined by the double bond for each isomer (Figure S10). Concerning **42a**, the sp^2^ methine proton δ H_11_ at 5.15 ppm showed a strong NOE connectivity to H_13_ and H_14_, while the H_3_ had a strong NOE cross peak to the allylic proton of the methyl group C_15_ at δ_C_ 16.21 ppm. This clearly indicated that the double bond in the side chain of **42a** has an E configuration. These spectral features require a structure of p-menth-1-en-3-[2-(E)-methylbut-1-enyl]-8-ol for **42**. Contrary, the double bond brought by the **42b** side chain was found as Z stereochemistry; these was assigned according to correlations between H_3_ → H_13_, and H_11_ → H_15_, respectively (Figure 5).

Both alcohol isomers **42a** and **42b** possess a humbertiane skeleton, a relatively rare sesquiterpene pattern. To our knowledge, only four isomeric isohumbertiols, structurally related to the alcohols **42a** and **42b,** were identified from the wood of *Humbertia madagascariensis Lam* [25]. In addition, two analogous structures were isolated after fungal transformation of α-farnesene while the NMR data (^1^H and ^13^C) reported in the literature [26], differed significantly from the experimental data described here for both new sesquiterpene alcohols.

### 2.3. P. porelloides Volatile Components: Chemical Compositions of Specific Plant Extracts

The chemical compositions of essential oil (EO), hydrosol extract (HY), both hexane and diethyl oxide extracts (EXT_H_ and EXT_O_), volatile fraction (VF) as well as microwave extract (MW) were investigated by GC/RI, GC-MS and ^13^C-NMR (Table 4).

*P. porelloides* EO was dominated by hydrocarbon compounds (49.4%), among them two sesquiterpenes were predominated: β-barbatene **27** (28.7%) and bicyclogermacrene **36** (8.2%). Oxygenated compounds were represented by 11 sesquiterpene alcohols (27.7%) and 10 non-terpenic compounds (0.7%). The other main components were globulol **50** (4.4%), viridiflorol **51** (5.8%) and maalian-5-ol **55** (5.4%). Hydrosol extract (HY) was dominated by globulol **50** (17.2%), maalian-5-ol **55** (9.4%), spathulenol **48** (9.3%) and rosifoliol **54** (7.3%). Relative to the essential oil, no hydrocarbon compounds were detected in the LLE extract obtained from hydrosol.

Unlike the essential oil, both hexane and diethyl oxide extracts (EXT_H_ and EXT_O_) were dominated by β-barbatene **27** (23.1 and 19.5%, respectively) and 1,2-dihydro-4,5-dehydronerolidol **58** (13.3 and 15.7%, respectively) which was not detected in the essential oil. Bicyclogermacrene **36** (16.4 and 14.1%, respectively), rosifoliol **54** (5.9 and 6.1%, respectively) and aristolene **23** (5.0 and 1.5%, respectively) were presents in remarkable amounts. The assisted microwave extract (MW) exhibited close chemical composition with β-barbatene **27** (32.6%), bicyclogermacrene **36** (17.8%) and 1,2-dihydro-4,5-dehydronerolidol **58** (17.2%), as main components. Finally, the volatiles emitted by the plant and sampled by SPME were β-barbatene **27** (39.5%), aristolene **23** (7.32%), bicyclogermacrene **36** (14.2%) and maalian-5-ol **55** (1.2%).

The volatile metabolites of *P. porelloides* were very atypical but the most surprising was the exclusive presence of an oxygenated linear sesquiterpene **58** in the solvent extracts. It is likely that hydrodistillation conditions cause the degradation of **58** into several compounds. However, the presence of **58** in the microwave extract proves that the temperature is not the only experimental parameter responsible for the degradation process. It is very likely that the protic character of water plays an important role. The absence of **58** in the volatile fraction emitted by the plant material can be explained by the conditions used for SPME; particularly the selectivity of the adsorbent phase seems to be implicated. The SPME experiment carried out on the plant extract support this hypothesis, allowing to detect the entire components previously listed except **58** [28].

It is difficult to accurately distinguish between the real essential oil constituents and those issued by the compound degradation. As the plant resources are limited, hemisynthesis trials become too complicated, especially as bicyclogermacrene is known to degrade into viridiflorol and spathulenol [29,30]. However, in our work, these compounds are present in samples of cold and hot extractions, we think that these compounds may be naturally present in *P. porelloides*.

### 2.4. Evaluation of Biological Activity: Antitrypanosomal, Antileishmanial and Cytotoxic Activities

To complete our study, we tested the antiparasitic activity of *P. porelloides* EO and EXT_O_ against two parasite models, *Leishmania mexicana mexicana* and *Trypanosoma brucei brucei*, respectively. In particular, *Leishmania mexicana mexicana* is responsible for leishmaniasis disease which drastically impacts the Corsican territory.

It could be noted that *P. porelloides* EO and EXT_O_ could be considered as having a good activity with IC50 values ≤ 20 µg/mL of *Leishmania mexicana mexicana* and the best activity (<6 µg/mL) on *Trypanosoma brucei brucei* [27]. Concerning the selectivity which was assessed here on the human non cancer fibroblast cell line WI38, only the essential oil had a sufficient selectivity (SI: 11.7) to be a good candidate for bioguided analysis.

Concerning cytotoxic activities, we observed that diethyl oxide extract of *P. porelloides* could be considered as having a good potential with IC_50_ value ≤ 5 µg/mL of WI38 and J774 (murine cancer macrophages) cells, but we did not find a clear selectivity on the cancer cell line. Nevertheless, this cytotoxicity may be interesting in the search for anticancer agents (Table 5).

It must be mentioned that earlier studies focused on different liverworts reported also an antiprotozoal activity, notably for alpha-eudesmol [31] and marchantin A [32].

## 3. Materials and Methods

### 3.1. Plant Material

Fresh *P. porelloides* were harvested in 2019 in one location of Corsica (France). Botanical determination was performed according to the determination keys summarized in Bryophyte Flora [33] and voucher specimens were deposited in the herbarium of University of Corsica, Corte (France).

### 3.2. Essential Oil and Hydrosol Isolation

After 15 days of drying, plant material (500 g) was subjected to hydrodistillation (HD) for 5 h using a *Clevenger*-type apparatus according to the method recommended in the *European pharmacopoeia* [34]. Hydrosol (300 mL) obtained by HD was submitted to Liquid/Liquid Extraction (LLE) in order to obtain a liquid extract (HY, 57.3 mg). LLE was performed three times using 50 mL of diethyl oxide.

The essential-oil yield (0.2%) was expressed in % (w/dw) based on the weight of the dried plant material.

### 3.3. SPME Experiments

Volatile fractions (VF) emitted by the plant were extracted with the HS-SPME method. Bryophytes samples (1 g) were crushed and disposed into 20 mL headspace vials. The vials were sealed with a silicon septum placed in 70 °C dry bath, and equilibrated for 30 min. A 75 μm DVB/CAR/PDMS solid-phase fiber (Supelco, Bellefonte, PA, USA) was then plugged into the headspace of the vials for 60 min. Later, the volatiles sampled by the solid-phase fiber were analyzed after desorption into the gas chromatography-mass spectrometry (GC-MS) injection port (5 min) in splitless mode. Each sample was conducted in triplicate.

### 3.4. Solvent Extractions

Dried plant materials (200 g) were mechanically powdered and extracted with hexane and diethyl oxide at room temperature for 24 h each in order to give after filtration and concentration in vacuum, both extracts called EXT_H_ (400 mg) and EXT_O_ (640 mg), respectively.

### 3.5. Microwave-Assisted Extractions

Dried plant materials were mechanically powdered and extracted using Multiwave 3000 (Anton Paar, Gratz, Austria) apparatus provided with 16 ceramic vessels. For each vessel, 5 g of dried bryophyte were introduced with 40 mL of hexane and extraction was realized at 180 °C during 20 min. The solvent was then filtered on activated carbon and concentrated under vacuum. The resulting extract was next taken up in absolute ethanol and centrifuged (20 min at 6000× *g* rpm) and the supernatant was collected and concentrated to finally obtain the MAE extract.

### 3.6. Essential Oil (EO) and Diethyl Oxide Extract (EXT_O_) Fractionations

Essential oil (EO, 800 mg) and diethyl oxide extract (EXT_O_, 640 mg) of *P. porelloides* was submitted to column chromatography (CC) on a silica-gel column (200–500 µm, 12 g, Clarisep^®^ Bonna Agela Technologies, Willington, NC, USA) with Combi Flash apparatus (Teledyne ISCO, Lincoln, NE, USA) equipped with a fraction collector monitored by an UV detector. Using gradients of (*v*/*v*) hexane/diisopropyl oxide (HEX/DIPO), forty-seven fractions (2 hydrocarbon fractions and 45 oxygenated fractions) were eluted from EO and two (one hydrocarbon fraction and one oxygenated fraction) were eluted from EXT_O_.

### 3.7. GC-FID Conditions

Analyses were carried out using a Perkin-Elmer Clarus 600 Gas Chromatography (GC) apparatus (Walthon, MA, USA) equipped with a single injector and two flame ionization detectors (FIDs) for simultaneous sampling to two fused-silica capillary columns (60 m × 0.22 mm i.d., film thickness 0.25 μm; Restek, Bellefonte, PA, USA) with stationary phases of different polarity, i.e., a nonpolar *Rtx-1* (polydimethylsiloxane) and a polar *Rtx-Wax* (polyethylene glycol). The oven temperature was programmed to rise from 60 to 230 °C at 2 °C min^−1^ and held isothermal at 230 °C for 30 min. The injector temperature was maintained at 280 °C and detector temperature at 280 °C, the carrier gas was H_2_ (0.7 mL.min^−1^) and the samples were injected (0.1 μL of pure oil) in the split mode (1:80). Retention indices (RIs) of the compounds were determined relative to the retention times (*t_R_*) of a series of n-alkanes (C5–C30; commercial solution obtained from *Restek*, Bellefonte, PA, USA) using the Van den Dool and Kraqtz equation [35].

### 3.8. GC-MS Analysis

Essential oils, extracts and fractions obtained by CC were investigated using a Perkin Elmer Turbo Mass quadrupole detector directly coupled to a Perkin Elmer Autosystem XL (Walton, MA, USA), equipped with the two same fused-silica capillary columns as described above. Both columns were used with the same MS detector. The analyses were consecutively carried out on the nonpolar and on the polar column. Hence, for each sample, two reconstructed ionic chromatograms (*RIC*) were provided, which were investigated consecutively. The GC conditions were the same as described above and the MS parameters as follows: ion-source temperature, 150 °C, ionization energy, 70 eV; electron ionization mass spectra acquired over a mass range of 35–350 amu during a scan time 1 s. The injection volumes for the essential oil and the fractions were 0.1 μL, and 0.2 μL, respectively.

### 3.9. High Resolution Mass Spectrometry Experiments

High resolution mass spectrometry experiments were performed with a Synapt G2 HDMS quadrupole/time-of-flight (Manchester, UK) equipped with an electrospray source operating in positive mode. Samples were introduced at 10 µL.min^−1^ flow rate (capillary voltage +2.8 kV, sampling cone voltage: varied between +20 V and +30 V) under a curtain gas (N_2_) flow of 100 L.h^−1^ heated at 35 °C. Accurate mass experiments were performed using reference ions from CH_3_COONa internal standard. The samples were dissolved and further diluted in methanol (Sigma-Aldrich, St-Louis—MO, USA) doped with formic acid (1% *v*/*v*) prior to analysis. Data analyses were conducted using MassLynx 4.1 programs provided by Waters.

### 3.10. NMR Conditions

Nuclear Magnetic Resonance (NMR) spectra were recorded on the CC-fraction **F27** obtained from the EO and the polar CC-fraction obtained from the EXT_O_. NMR experiments were acquired in CDCl_3_ (EuroIsotop, Saint Aubin, France), at 300 K using a Bruker Avance DRX 500 NMR spectrometer (Karlsruhe, Germany) operating at 500.13 MHz for ^1^H and 125.77 MHz for ^13^C Larmor frequency with a double resonance broadband fluorine observe (BBFO) 5 mm probe head. ^13^C-NMR experiments were recorded using one-pulse excitation pulse sequence (90° excitation pulse) with ^1^H decoupling during signal acquisition (performed with WALTZ-16); the relaxation delay was set at 2 s. For each analyzed sample, depending on the compound concentration, 3 k up to 5 k free induction decays (FID) 64 k complex data points were collected using a spectral width of 30,000 Hz (240 ppm). Chemical shifts (δ in ppm) were reported relative to residual signal of CDCl_3_ (δ_C_ 77.04 ppm). Complete ^1^H and ^13^C assignments of the new compound were obtained using 2D gradient-selected NMR experiments, ^1^H-^1^H COSY (COrrelation SpectroscopY), ^1^H-^13^C HSQC (Heteronuclear Single Quantum Correlation), ^1^H-^13^C HMBC (Heteronuclear Multiple Bond Coherence) and ^1^H-^1^H NOESY (Nuclear Overhauser Effect SpectroscopY), for which conventional acquisition parameters were used, as described in the literature [36].

### 3.11. Identification of Components

The identification of individual components in essential oil, extracts or CC-fractions was based on a methodology involving integrated techniques, such as GC retention indices, GC-MS (EI) and NMR. Identification of volatiles sampled by SPME were carried only by GC retention indices and GC-MS (EI). The identification of individual components was based (i) on the comparison of the retention indices (*RIs*) determined on the polar and nonpolar columns with those of authentic compounds or literature data [27,37] (ii) on computer matching of the mass spectra with commercial MS-libraries and the mass spectra with those listed in our homemade MS-library built of mass spectra of authentic compounds or literature data [38,39] (iii) comparing the ^13^C-NMR chemical shifts of CC-fraction components with those of reference spectra reported in the literature (iv) NMR assignments using 1D and 2D data.

1,2-dihydro-4,5-dehydronerolidol **58**: yellow oil (20 mg); RI apolar 1616, RI polar 2136; for ^1^H and ^13^C NMR data (see Table 2); MS (EI; 70 eV) *m*/*z* (rel. Int): 222 [M+] (4), 107 (100), 41 (80), 135 (73), 69 (62), 91 (53), 93 (53), 105 (49), 43 (46), 79 (40), 204 (30), 119 (30), 161 (29), 77 (28), 55 (26). HRMS: detected ion *m*/*z* 245.1879 [MNa^+^] (calc. for C_15_H_26_ONa^+^, error: +1.2 ppm).

*p*-menth-1-en-3-[2-methylbut-1-enyl]-8-ol **42a,b**: colorless oil (7 mg); RI apolar 1528, RI polar 1917; for ^1^H and ^13^C NMR data (see Table 3); MS (EI; 70 eV) *m*/*z* (rel. Int): 222 [M+] (1), 107 (100), 59 (31), 161 (31), 91 (31), 41 (28), 175 (26), 105 (25), 135 (23), 79 (23), 119 (22), 93 (21), 108 (19), 43 (16), 77 (16). HRMS: detected ion *m*/*z* 245.1878 [MNa^+^] (calc. for C_15_H_26_ONa^+^, error: +0.8 ppm).

### 3.12. Component Quantification

The quantification of components was performed using the methodology reported by Bicchi [40] and adapted in our laboratory [41]. Briefly, the compound quantification was carried out using peak normalization, including FID response factors relative to tridecane (0.7 g/100 g) used as internal standard, and expressed as normalized contents (% abundances).

### 3.13. Parasites, Cells and Media

*Trypanosoma brucei brucei* (strain 427) bloodstream forms were cultured in vitro in HMI9 medium containing 10% heat-inactivated fetal bovine serum [42]. *Leishmania mexicana mexicana* promastigotes (MHOM/BZ/84/BEL46) were cultivated in vitro in a semi-defined medium (SDM-79) [43] supplemented with 15% heat-inactivated fetal bovine serum. The human normal fibroblast cell line, WI-38, was cultivated in vitro in DMEM medium containing 4 mM L-glutamine, 1 mM sodium pyruvate supplemented with 10% heat-inactivated fetal bovine serum and penicillin–streptomycin (100 UI/mL to 100 µg/mL). All cells were incubated in a humidified atmosphere with 5% CO_2_ at 37 °C except the *Leishmania promastigotes* which were incubated at 28 °C.

### 3.14. In Vitro Test for Antitrypanosomal and Antileishmanial Activity

The in vitro test was performed as described previously [44]. Pentamidine isethionate salt (a commercial antileishmanial drug) and suramine sodium salt (a commercial antitrypanosomal drug) were used as positive controls in all experiments with an initial concentration of 10 µg/mL. First, stock solutions of crude extracts and compounds were prepared in DMSO at 20 mg/mL (and 2 mg/mL for positive controls). The solutions were further diluted in medium to give 0.2 mg/mL stock solutions. Essential oil and extracts were tested in eight serial three-fold dilutions (final concentration range: 100–0.05 µg/mL) in 96-well microtiter plates. All tests were repeated three times in duplicate.

### 3.15. Cytotoxicity Assay

The cytotoxicity of the essential oil and extracts on WI-38 and J774 cells was evaluated as previously described from the same stock solutions [45].

## 4. Conclusions

Three new phytochemicals were isolated and identified from Corsican *Plagiochila porelloides*. Both (E) and (Z) stereoisomers showing an unusual humbertiane skeleton, namely p-menth-1-en-3-[2-methylbut-1-enyl]-8-ol, could be fully characterized using combined analytical approach. Moreover, an atypic aliphatic compound, named 1,2-dihydro-4,5-dehydronerolidol is also newly reported and fully characterized in this work. The in-vitro antiprotozoal activity of essential oil and extract of *P. porelloides* against *Trypanosoma brucei brucei* and *Leishmania mexicana mexicana* and cytotoxicity were assessed. Essential oil and diethyl oxide extract showed a moderate activity against *T. brucei* (IC50 values found were 2.03 and 5.18 μg/mL, respectively). It is noteworthy that only the essential oil sample has shown a high selectivity (SI = 11.7), whereas the diethyl oxide extract exhibited moderate anticancer (cancerous macrophage-like murine cells) activity and cytotoxicity (human normal fibroblast) with IC_50_ values: 1.25 and 2.96 μg/mL, respectively.

## Figures and Tables

**Table 1:** Summary of principal volatile molecules reported for essential oils of different *Plagiochila* species [13,14,15,16].

Structure of the Main Volatile Compounds		
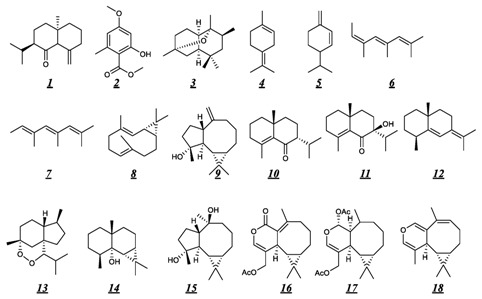		
Species	Hydrocarbon terpenes	Oxygenated terpenes
*P. biffaria* [13]		(-)-5R,7R,10S-eudesm-4(15)-en-6-one (9–19%, ***1***) Methyl everninate (1–35%, ***2***) peculiaroxide (13–16%, ***3***)
*P. madernensis* [13]	terpinolene (34–60%, ***4***)	
*P. retrorsa* [13]	β-phellandrene (16–46%, ***5***)	peculiaroxide (9–12%, ***3***)
*P. retrorsa* [13]	*allo*-ocimene (15%, ***7***) *neo*-*allo*-ocimene (10%, ***6***) terpinolene (13%, ***4***)	peculiaroxide (12%, ***3***)
*P. stricta* [13]	*allo*-ocimene (7–19%, 7) bicyclogermacrene (4–17%, ***8***) *neo*-*allo*-ocimene (4–11%, ***6***)	peculiaroxide (11–21%, **3**) Spathulenol (2–14%, ***9***)
P. biffaria * [14]		ent-eudesm-4-en-6-one (***10***) ent-eudesm-4(15)-en-6-one (***1***) ent-7-hydroxyeudesm-4-en-6-one (***11***)
*P. asplenioides* [15]	(-)-selina-5,7(11)-diene (8%, ***12***)	plagio-4,7-peroxide (20%, ***13***) maalian-5-ol (19%, ***14***)
P. ovalifolia * [16]		ent-4ß,10α-dihydroxyaromadendrane (***15***) Acetoxyisoplagiochilide (***16***) Maalian-5-ol (***13***) plagiochiline C (***17***) Plagiochiline N (***18***)

* Corresponding percentages are not indicated.

**Table 2:** Full NMR data of 1,2-dihydro-4,5-dehydronerolidol (**58**) (500 MHz, 300 K and CDCl_3_).

Atom No.	^13^C NMR		^1^H NMR			^1^H–^1^H and ^1^H–^13^C 2D Correlations		
	δ (ppm)	Type	δ (ppm)	Mult	J (Hz)	HMBC *	NOESY	COSY
1	8.37	CH_3_	0.9	t	7.4	H_2_	H_2_	-
2	35.43	CH_2_	1.6	dd	7.44 & 1.6	H_1_ H_13_	H_1_ H_13_	-
3	73.38	C-O	-	-	-	H_1_ H_2_ H_4_ H_5_ H_13_	-	-
4	137.80	CH=	5.6	d	15.3	H_2_ H_6_ H_13_	H_6_ H_13_	H_6_
5	123.99	CH=	6.5	dd	15.3 & 10.8	H_6_ H_13_	H_13_ H_14_	H_14_
6	124.13	CH=	5.9	d	10.8	H_4_ H_5_ H_8_ H_14_	H_8_/H_9_	H_8_/H_9_
7	138.75	C=	-	-	-	H_5_ H_8_ H_14_	-	-
8	39.97	CH_2_	2.1	m	-	H_9_ H_14_	H_6_	-
9	26.62	CH_2_	2.1	m	-	H_8_	H_10_ H_14_ H_15_	H_14_ H_15_
10	124.03	CH=	5.1	t	6.0	H_8_ H_12_ H_15_	H_9_ H_12_ H_15_	H_12_
11	131.71	C=	-	-	-	H_12_ H_15_	-	-
12	25.71	CH_3_	1.7	s	-	H_10_ H15	H_10_	-
13	27.58	CH_3_	1.3	s	-	H_4_	H_1_	-
14	16.75	CH_3_	1.8	s	-	H_6_	H_5_	-
15	17.70	CH_3_	1.6	s	-	H_10_ H_12_	H_10_	-

* HMBC correlation are reported as C->H.

**Table 3:** Full NMR data of *p*-menth-1-en-3-[2-methylbut-1-enyl]-8-ol isomers **42a** and **42b** (in CDCl_3_, at 500 MHz and 300 K)**.**

Atom No.	42a						42b					
	^13^C NMR		^1^H NMR		2D NMR		^13^C NMR		^1^H NMR		2D NMR	
	δ (ppm)	DEPT	δ (ppm)	Mult (J, Hz)	HMBC *	NOESY	δ (ppm)	DEPT	δ (ppm)	Mult (J, Hz)	HMBC *	NOESY
1	134.16	C	-	-	H_7_		134.29	C	-	-	H_7_	
2	124.88	CH	4.95	d (1.26)	H_7_	H_3_ H_7_	125.19	CH	4.95 s	d (1.26)	H_7_	H_3_ H_7_
3	37.18	CH	3.08	m		H_2_ H_5_ H_9_ H_11_ H_15_	36.93	CH	3.08	m	H_5_	
4	50.48	CH	1.5	m	H_11_ H_2_ H_10_ H_9_	H_5_ H_6_	50.54	CH	1.34	m	H_9_ H_10_	H_5_ H_6_
5	25.37	CH_2_	1.83/1.31	m	H_3_ H_4_ H_6_		25.29	CH_2_	1.83/1.31	m		
6	30.7	CH_2_	2.03	m	H_2_ H_7_		30.65	CH_2_	1.96	m	H_5_ H_7_	H_5_
7	23.31	CH_3_	1.67	s	H_2_	H_6_	23.33	CH_3_	1.64	s		H_7_
8	74.38	C-OH	-	-	OH H_10_ H_9_		74.38	C-OH	-	-	H_9_ H_10_	
9	25.78	CH_3_	1.17	s	H_10_	H_3_	25.71	CH_3_	1.17	s	H_10_	H_3_
10	28.93	CH_3_	1.24	s	OH H_9_	H_4_ H_5_	28.99	CH_3_	1.23	s	H_8_ H_9_	H_4_ H_5_
11	129.59	CH	5.15	dd (10.1 and 1.26)	H_13_ H_15_	H_4_ H_6_ H_13_ H_14_ OH	130.69	CH	5.11	d(10.1)	H_13_ H_15_	H_15_
12	137.34	C	-	-	H_13_ H_15_ H_14_		137.4	C	-	-	H_13_ H_14_ H_15_	
13	32.5	CH_2_	2.03	q (7.32)	H_11_ H_14_ H_15_	H_14_ H_15_	25.11	CH_2_	2.19/2.09	m	H_14_ H_15_	H_3_ H_14_
14	12.55	CH_3_	1.02	t (7.32)	H_13_	H_13_	12.85	CH_3_	1.06	t (7.23)	H_13_	H_13_
15	16.21	CH_3_	1.73	d (0.88)	H_11_ H_13_	H_3_ H_9_ H_13_ H_14_	22.98	CH_3_	1.74	d (1.47)	H_11_ H_13_	H_3_ H_9_ H_13_ H_14_
		OH	2.6	OH				OH	2.6	OH		

* HMBC correlations are reported as C->H.

**Table 4:** Chemical compositions of *P. porelloides* samples from Corsica.

					Samples ^5^							
No ^1^	Compounds	L RI_A_ ^2^	RI_A_ ^3^	RI_P_ ⁴	EO	HY	EXT		MW	VF	Reference ^6^	
							EXT_H_	EXT_O_				
**1**	hexanal	770	771	819	t	-	t	t	-	t	IR, MS	
**2**	heptanal	876	876	1079	t	-	t	t	-	t	IR, MS	
**3**	α-pinene	931	931	1032	t	-	t	t	-	1.0	IR, MS	
**4**	camphene	943	944	1066	t	-	t	t	-	0.1	IR, MS	
**5**	6-methylhept-5-en-2-one	963	954	1343	0.1	-	t	-	-	0.1	IR, MS	
**6**	oct-1-en-3-ol	959	962	1453	0.4	1.9	-	t	-	t	IR, MS	
**7**	octen-3-one	963	980	1260	t	-	-	t	-	0.1	IR, MS	
**8**	octan-3-ol	982	982	1401	t	-	-	t	-	-	IR, MS	
**9**	phenylaldehyde	1013	1010	1616	t	-	-	-	-	t	IR, MS	
**10**	β-phellandrene	1021	1021	1219	t	-	-	0.1	-	0.4	IR, MS	
**11**	nonanal	1083	1083	1396	0.1	-	t	-	-	-	IR, MS	
**12**	octen-3-yl acetate	1094	1094	1377	0.1	-	t	0.6	0.2	2.4	RI, MS, Ref	
**13**	decanal	1186	1185	1496	t	-	-	-	-	-	RI, MS, Ref	
**14**	bicycloelemene	1334	1334	1552	0.3	-	0.9	1.1	0.4	2.0	RI, MS, Ref	
**15**	maali-1,3-diene	1347	1346	1532	0.8	-	0.5	0.6	-	1.5	RI, MS, Ref	
**16**	anastrepene	1373	1369	-	0.2	-	0.4	1.2	0.8	1.4	RI, MS, Ref	
**17**	isoledene	1372	1373	-	0.1	-	0.1	0.2	-	0.2	IR, MS	
**18**	α-copaene	1379	1376	1498	0.2	-	0.4	0.4	-	1.7	IR, MS	
**19**	β-elemene	1388	1388	1592	0.2	-	0.4	0.6	-	1.4	IR, MS	
**20**	african-3-ene	1391	1390	-	-	-	0.0	0.3	-	1.1	RI, MS, Ref	
**21**	α-barbatene	1414	1409	1565	1.8	-	1.3	2.5	0.4	4.7	RI, MS, Ref	
**22**	tritomarene	1416	1413	1410	0.2	-	-	0.5	-	1.1	RI, MS, Ref	
**23**	**aristolene**	1420	1419	1581	2.6	-	5.0	1.5	4.1	7.3	RI, MS, NMR	
**24**	γ-maaliene	1428	1425	1613	1.2	-	2.9	0.2	3.0	0.5	RI, MS, Ref	
**25**	calarene	1439	1438	1603	0.6	-	0.5	0.6	0.5	1.0	IR, MS	
**26**	α-maaliene	1440	1440	-	0.5	-	t	0.6	0.8	-	RI, MS, Ref	
**27**	**β-barbatene**	1445	1445	1663	28.7	-	23.1	19.5	32.6	39.5	RI, MS, NMR	
**28**	aromadendrene	1447	1458	1601	0.3	-	0.4	0.5	-	0.6	RI, MS, Ref	
**29**	α-acoradiene	1464	1460	-	0.6	-	0.2	0.3	-	0.9	RI, MS, Ref	
**30**	β-acoradiene	1465	1462	-	0.1	-	0.4	0.2	-	0.4	RI, MS, Ref	
**31**	γ-curcumene	1475	1471	-	0.1	-	0.7	0.5	-	0.4	RI, MS, Ref	
**32**	β-chamigrene	1478	1473	-	1.3	-	0.5	0.8	-	1.5	RI, MS, Ref	
**33**	α-curcumene	1473	1481	1738	0.1	-	0.1	0.4	-	0.7	RI, MS, Ref	
**34**	β-selinene	1483	1485	-	1.1	-	0.0	0.1	-	-	RI, MS, Ref	
**35**	β-maaliene	1480	1481	-	0.1	-	0.1	0.3	-	-	RI, MS, Ref	
**36**	**bicyclogermacrene**	1494	1494	1744	8.2	-	16.4	14.1	17.8	14.2	RI, MS, NMR	
**37**	204[M]^+^; 107(100); 93(90)	1498	1497	-	1.5	-	0.4	0.9	-	1.0		
**38**	ledene	1494	1498	1706	t	-	-	0.9	-	0.4	RI, MS	
**39**	α-chamigrene	1503	1500	-	t	-	-	0.4	-	0.4	RI, MS, Ref	
**40**	γ-cadinene	1507	1514	1775	t	-	-	0.6	-	0.7	RI, MS	
**41**	α-alaskene	1512	1513	-	t	-	-	0.5	-	0.4	RI, MS, Ref	
**42a**	***p*-menth-1-en-3-[2-methyl-1E-butenyl]-8-ol**	-	1536	1917	1.2	3.4	0.3	1.2	-	-	RI, MS, NMR	
**42b**	***p*-menth-1-en-3-[2-methyl-1Z-butenyl]-8-ol**	-	1536	1917	3.8	-	0.6	-	-	-	RI, MS, NMR	
**43**	tamariscol	1535	1536	1917	1.2	-	0.6	1.1	-	-	RI, MS	
**44**	204[M]^+^; 161(100); 91(56)	-	1532	1917	3.7	-	-	-	-	-		
**45**	**4-epi-maaliol**	-	1544	-	0.7	-	0.6	0.3	-	-	RI, MS, NMR	
**46**	222[M]^+^; 107(100); 135(51)	-	1545	1832	0.9	4.7	-	-	-	-	-	
**47**	pallustrol	1567	1558	1923	t	-	-	t	-	-	RI, MS, Ref	
**48**	**spathulenol**	1557	1561	2090	0.7	9.3	1.1	0.6	3.5	-	RI, MS, NMR	
**49**	204[M]^+^; 107(100); 135(56)	-	1567	-	0.9	-	-	-	-	-	-	
**50**	**globulol**	1571	1578	2077	4.4	17.2	1.9	1.3	0.2	0.9	RI, MS, NMR	
**51**	**viridiflorol**	1591	1585	2085	5.8	4.2	1.9	-	-	0.3	RI, MS, NMR	
**52**	222[M]^+^; 107(100)	-	1588	-	1.2	-	-	-	-	-	-	
**53**	238[M]^+^; 149(100)	-	1591	1920	5.21	-	-	-	-	-	-	
**54**	**rosifoliol**	1599	1587	2108	3.8	7.3	5.9	6.1	0.4	-	RI, MS, NMR	
**55**	**maalian-5-ol**	1607	1595	2051	5.4	9.4	2.2	1.9	7.6	1.2	RI, MS, NMR	
**56**	204[M]^+^;107(100);135(76)	-	1611	1938	1.4	-	-	-	-	-	-	
**57**	222;107(100);105(75)	-	1613	-	1.3	4.2	-	-	-	-	-	
**58**	**1,2-dihydro-4,5-dehydronerolidol**	-	1616	2136	-	-	13.3	15.7	17.2	-	RI, MS, NMR	
	Total identified				76.9	52.6	82.6	77.9	89.4	90.3		
	Classes of compounds (%)											
	Hydrocarbon compounds				49.3	-	54.4	49.2	60.4	85.4		
	Oxygenated compounds				27.5	72.3	28.1	28.7	29.1	4.9		
	Monoterpene hydrocarbons				-	-	-	0.1	-	1.5		
	Monoterpene oxygenated				-	-	-	-	-	-		
	Sesquiterpene hydrocarbons				49.3	-	54.4	49.1	60.4	83.8		
	Sesquiterpene oxygenated				26.9	70.5	28.1	28.1	28.8	2.4		
	Other				0.6	1.9	-	0.6	0.2	2.5		

^1^ The order to elution is given in the apolar column (Rtx-1) ^2^ LRI_A_: literature retention indices on apolar column reported from the literature [27]. ^3^ RI_A_: retention indices on Rtx-1 (apolar) column ^4^ RI_P_: retention indices on Rtx-Wax (polar) column ^5^ Percentages of individual components on Rtx-1 except those with the same RI_A_; percentages given on Rtx-Wax column. ^6^ RI: retention indices; Ref: compounds identified from commercial libraries [27]; EO: essential oil obtained by hydrodistillation; HY: LLE extract obtained from hydrosol; EXT_H_ and EXT_O:_ hexane and diethyl oxide extracts (cold maceration); MW: assisted microwave extract; VF: volatiles sampled by SPME.

**Table 5:** The cytotoxicity (W138 and J774) and in-vitro activity of *P. porelloides* EO and EXT_O_ against *Leishmania mexicana mexicana* (Lmm) and *Trypanosoma brucei brucei* (Tbb).

Sample	Cytotoxicity		Antiprotozoal Assay		Selectivity Indices		
	IC_50_ ± SD in µg/mL (µM for Pure Compound)				SI = IC_50 (WI38)_/IC_50 (J774 or parasite)_		
	WI38	J774	Lmm	Tbb	J774	Lmm	Tbb
EO	23.85 ± 4.39	28.81 ± 0.45	15.99 ± 0.85	2.03 ± 0.12	0.8	1.5	11.7
EXT_O_	2.96 ± 0.17	1.25 ± 0.08	17.73 ± 1.14	5.18 ± 0.81	2.4	0.2	0.6
Camptothecin	0.031 ± 0.002	0.01 ± 0.001					
Pentamidine			0.07 ± 0.004				
Suramine				0.03 ± 0.004			

WI38: non-cancer human fibroblasts; J774: cancerous macrophage-like murine cells; Tbb: *Trypanosoma brucei brucei* (bloodstream forms); Lmm: *Leishmania mexicana mexicana* promastigotes; selectivity index calculated for antiparasitic activities compared to WI38 cytotoxicity.

## Data Availability

Not applicable.

## Supplementary Materials

The following supporting information can be downloaded at: https://www.mdpi.com/article/10.3390/molecules28020616/s1: Figure S1: ^1^H spectrum **58**, Figure S2: ^13^C -spectrum of **58**, Figure S3: ^1^H-^13^C HSQC spectrum of **58**; Figure S4: ^1^H-^13^C HMBC spectrum of **58**; Figure S5: ^1^H-^1^H NOESY spectrum of **58**; Figure S6: ^1^H spectrum of **42a** and **42b**; Figure S7: ^13^C spectrum of **42a** and **42b**; Figure S8: ^1^H-^13^C HMBC spectrum of **42a** and **42b**; Figure S9: ^1^H-^13^C HMBC spectrum of **42a** and **42b**; Figure S10: ^1^H-^1^H NOESY spectrum of **42a** and **42b**

## Abbreviation

EO: Essential Oil; GC: Gas Chromatography; FID: Flame Ionization Detector; NMR: Nuclear Magnetic Resonance; EI: Electron Impact; ESI: Electrospray Ionization; EXT_H_: hexane solvent extract; EXT_O_: diethyl oxide extract; MS: Mass Spectrometry; RI: retention Index; HRMS: High Resolution MS; SPME: Solid Phase MicroExtraction; HY: Hydrosol extract; MW: Microwave assisted extraction; VF: Volatile Fraction.
