# Supplementary material for: First Phytochemical Profiling and In-Vitro Antiprotozoal Activity of Essential Oil and Extract of Plagiochila porelloides
Source: Molecules. 2023 Jan 7;28(2):616. doi: 10.3390/molecules28020616 (PMC9860869; doi:10.3390/molecules28020616)
Supplement: Supplementary file 1 [file molecules-28-00616-s001.zip › molecules-2095133-supplementary.pdf]

First phytochemical profiling and in-vitro antiprotozoal activity of essential oil and extract of *Plagiochila porelloides*

Anaïs Pannequin[1], Joëlle Quetin-Leclercq[2], Jean Costa[1], Aura Tintaru[3*] and Alain Muselli[1*]

[1] Université de Corse, UMR CNRS 6134, Laboratoire Chimie des Produits Naturels, BP 52, 20250 Corte, France

[2]Louvain Drug Research Institute, UCLouvain, UCL 7230, Avenue E. Mounier 72, B-1200 Brussels, Belgium

[3] Aix Marseille Univ, CNRS, Centre Interdisciplinaire de Nanoscience de Marseille, UMR7325, 13288 Marseille, France

* Correspondence: muselli_a@univ-corse.fr; aura.tintaru@univ-amu.fr

Supplementary Material

Figure S1: ^{1}H spectrum **58**……………………………………………………………………2

Figure S2: ^{13}C spectrum of **58**……………………………………………………………………3

Figure S3: ^{1}H-^{13}C HSQC spectrum of **58**……………………………………………………………4

Figure S4: ^{1}H-^{13}C HMBC spectrum of **58**……………………………………………………………5

Figure S5: ^{1}H-^{1}H NOESY spectrum of **58**……………………………………………………………6

Figure S6: ^{1}H spectrum of **42a** and **42b**……………………………………………………………7

Figure S7: ^{13}C spectrum of **42a** and **42b**……………………………………………………………8

Figure S8: ^{1}H-^{13}C HSQC spectrum of **42a** and **42b**………………………………………………9

Figure S9: ^{1}H-^{13}C HMBC spectrum of **42a** and **42b**………………………………………………10

Figure S10: ^{1}H-^{1}H NOESY spectrum of **42a** and **42b**……………………………………………11

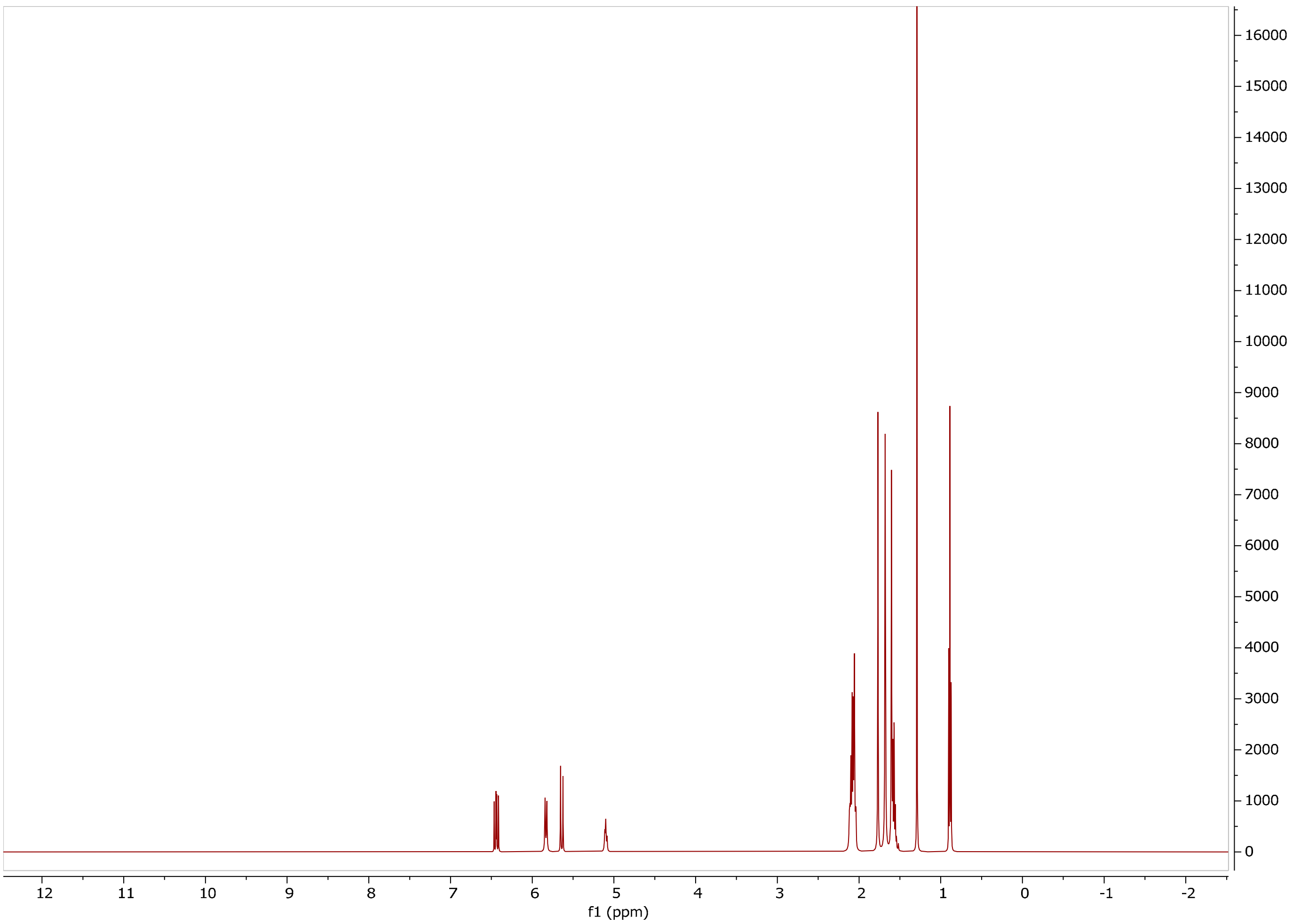

*Figure S1 ¹H-spectrum of **58***

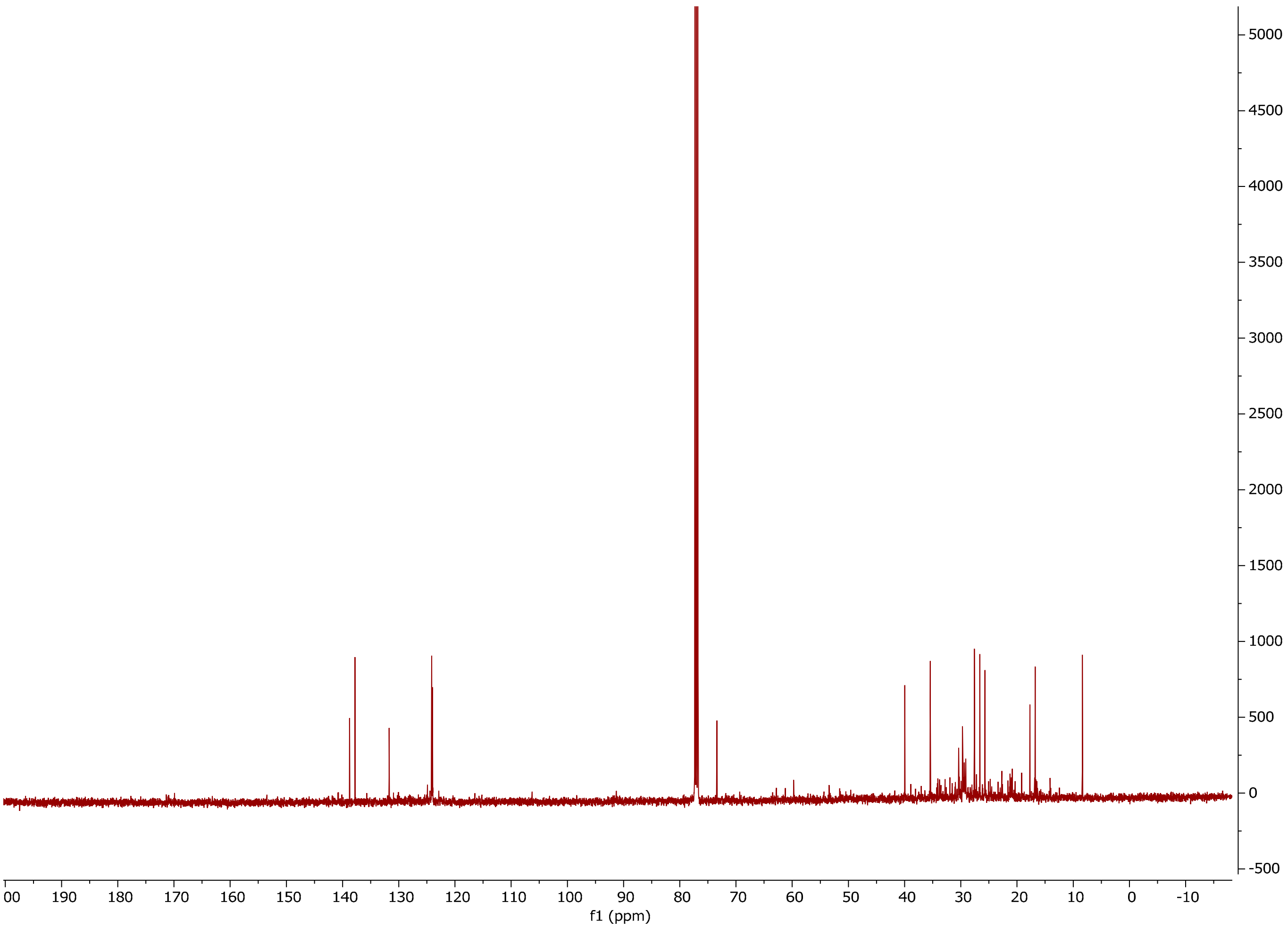

Figure S2 : ^{13}C -spectrum of [58]

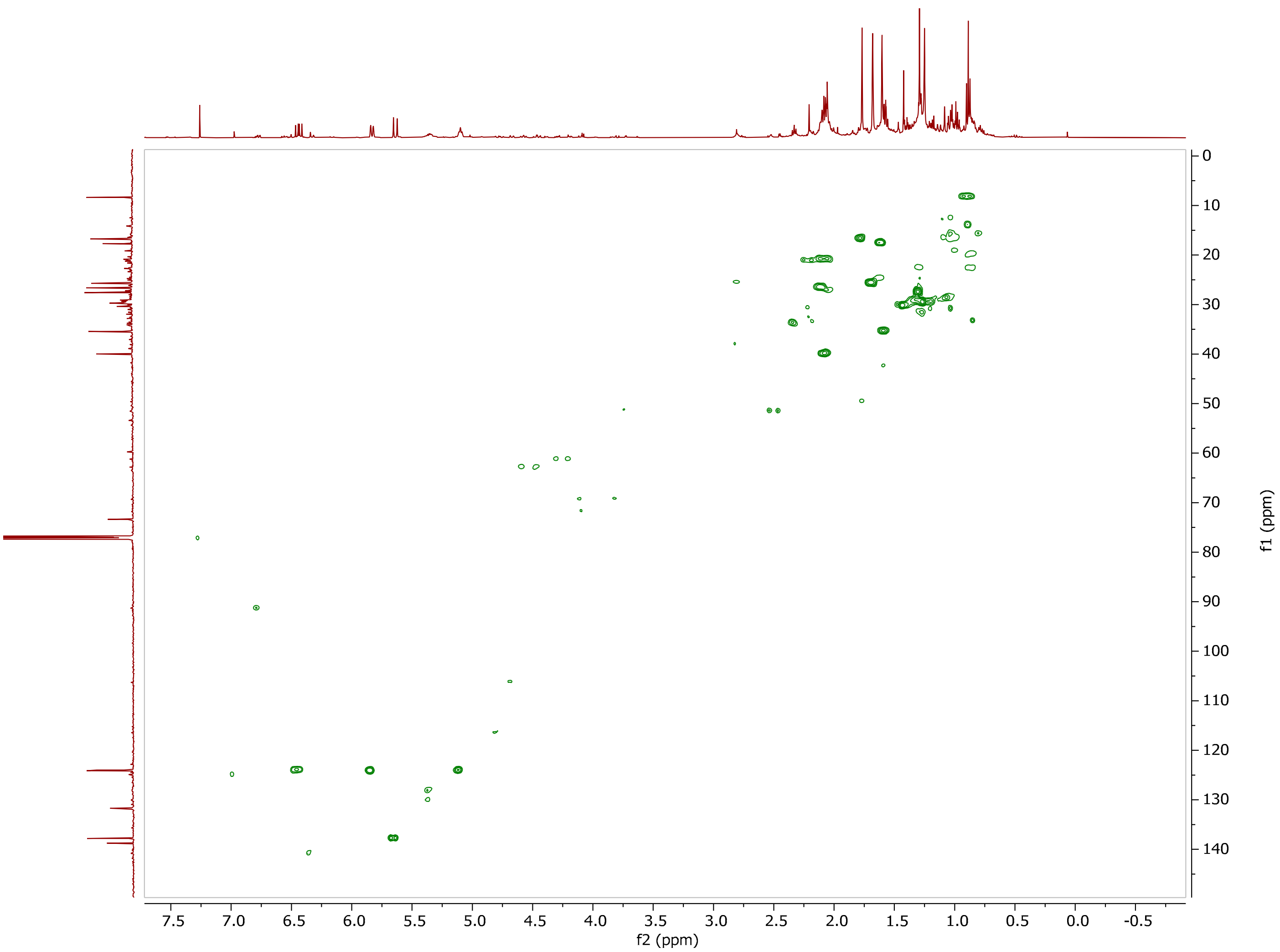

Figure S3 ^{1}H-^{13}C HSQC spectrum of [58]

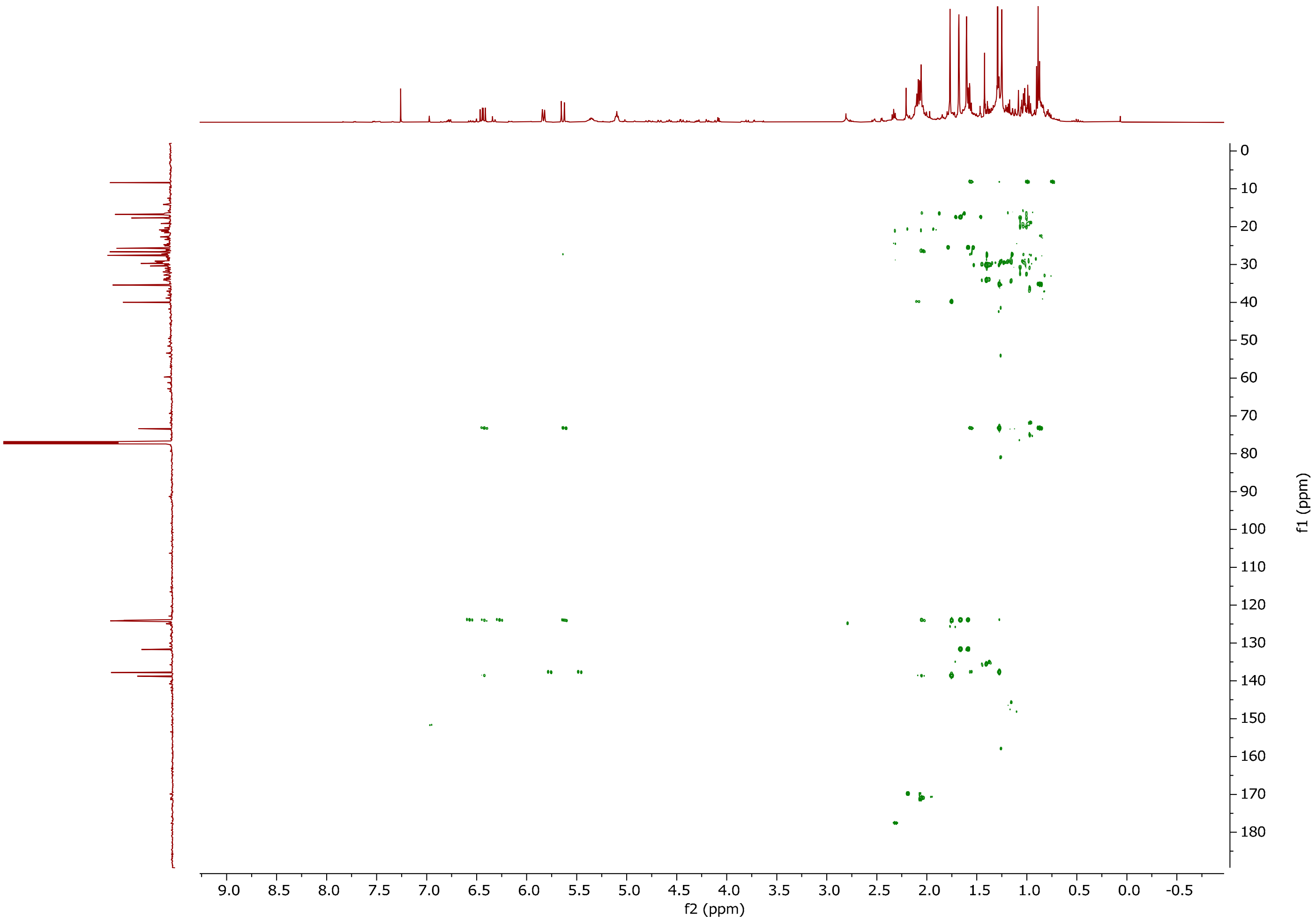

Figure S4 ^{1}H-^{13}C HMBC spectrum of ***58***

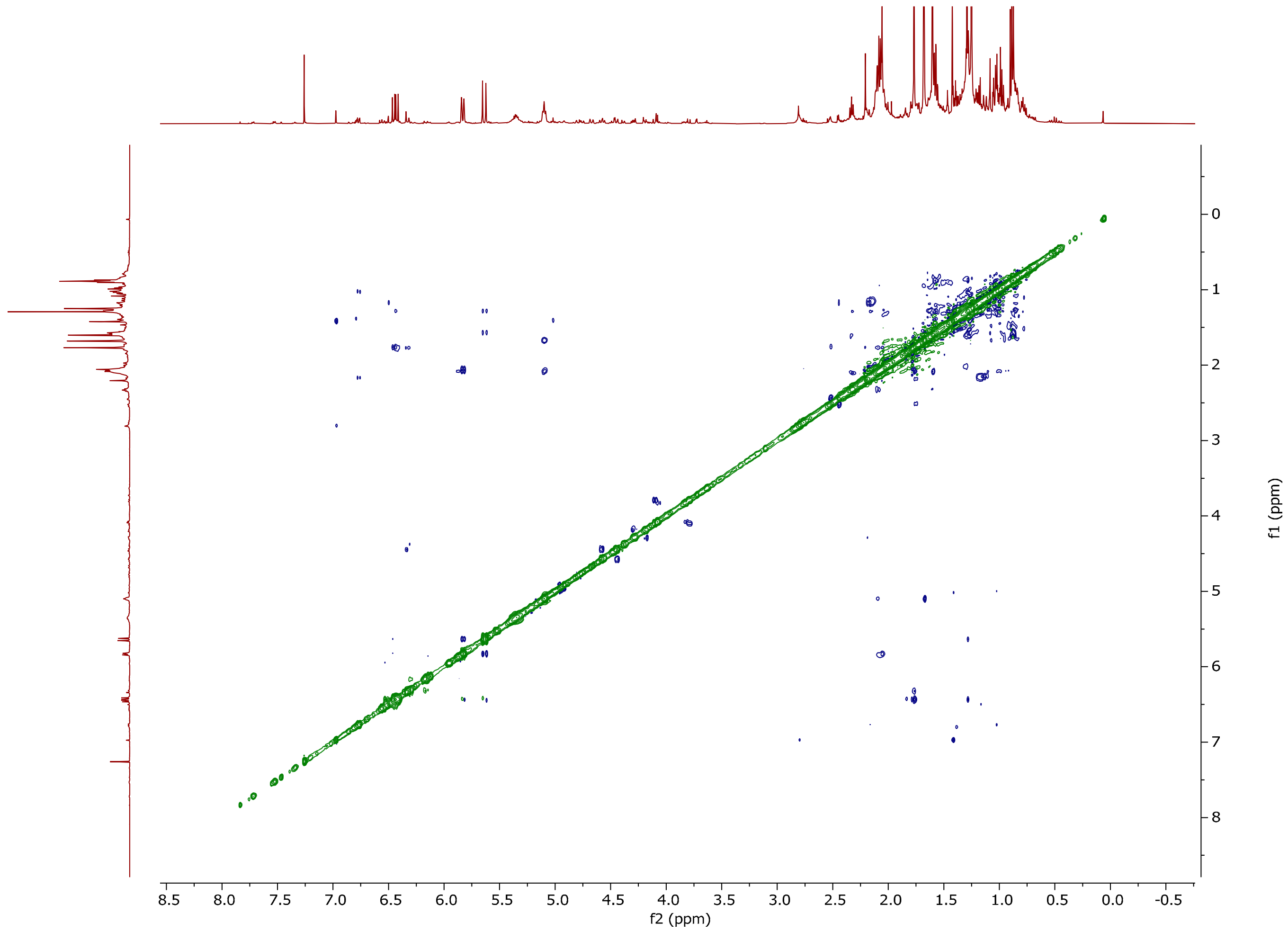

*Figure S5 ^{1}H-^{1}H NOESY spectrum of **58***

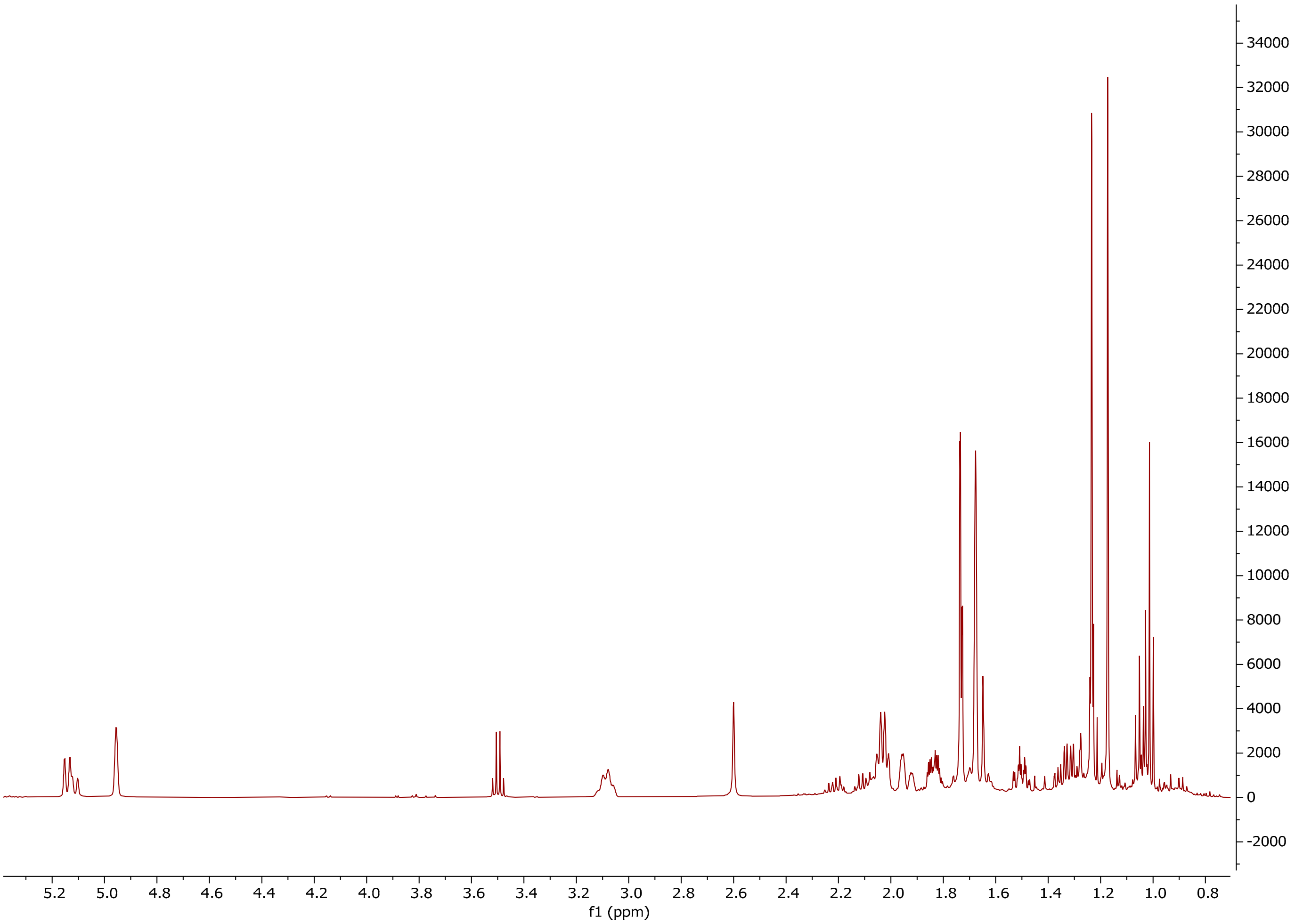

*Figure S6 ^1H spectrum of **42a** and **42b***

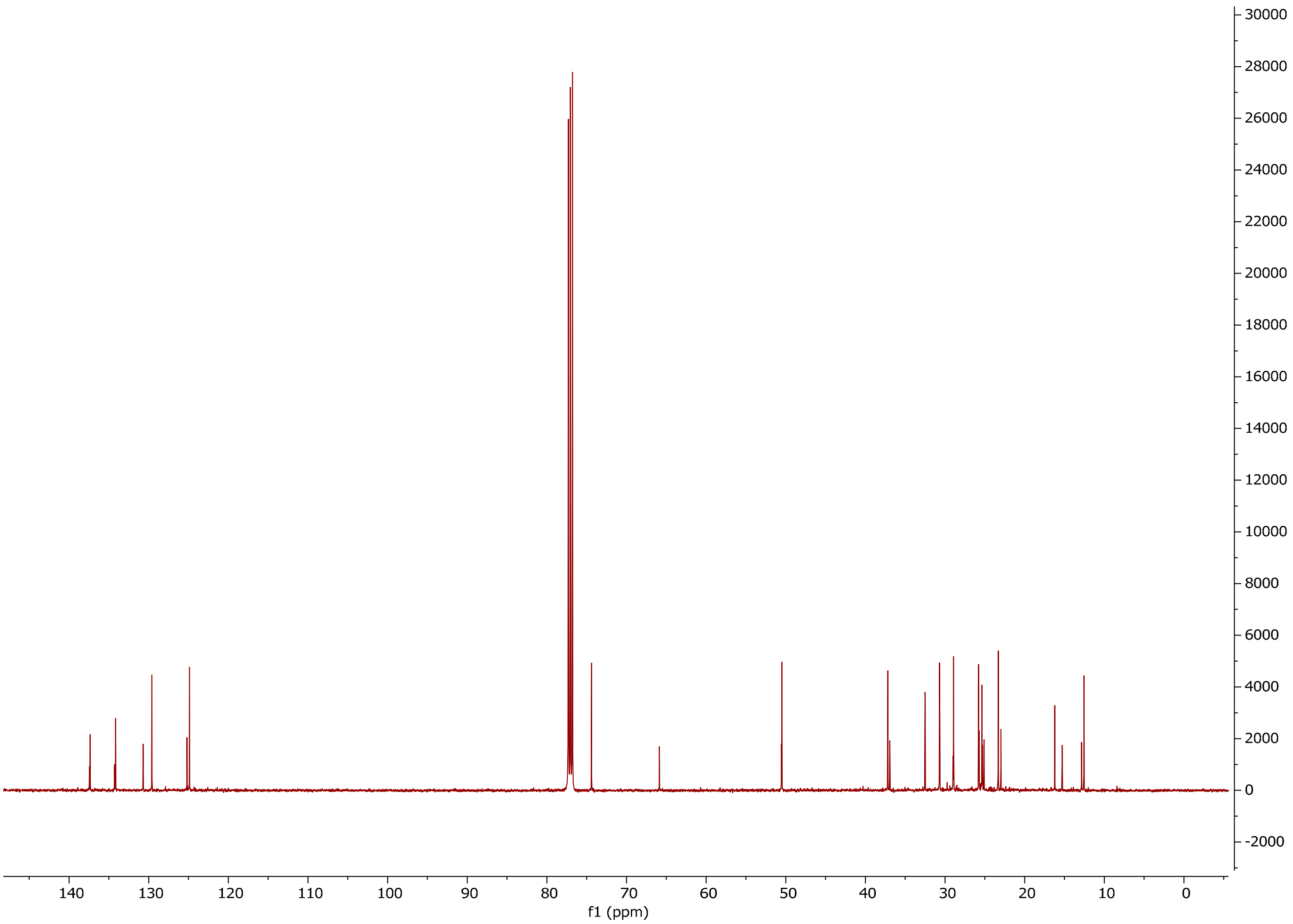

Figure S7 ¹³C spectrum of ***42a*** *and* ***42b***

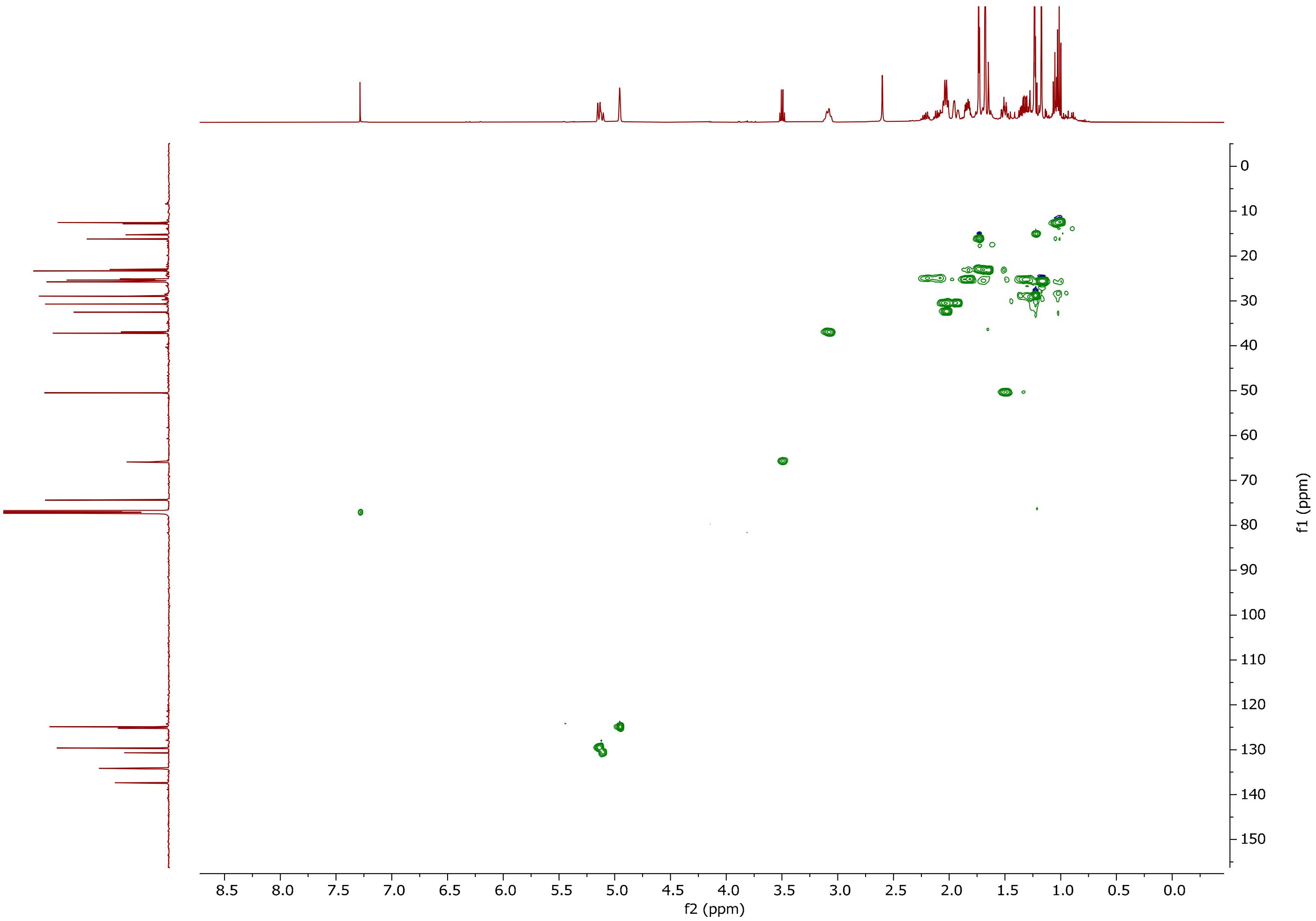

*Figure S8 ^{1}H-^{13}C HMBC spectrum of **42a** and **42b***

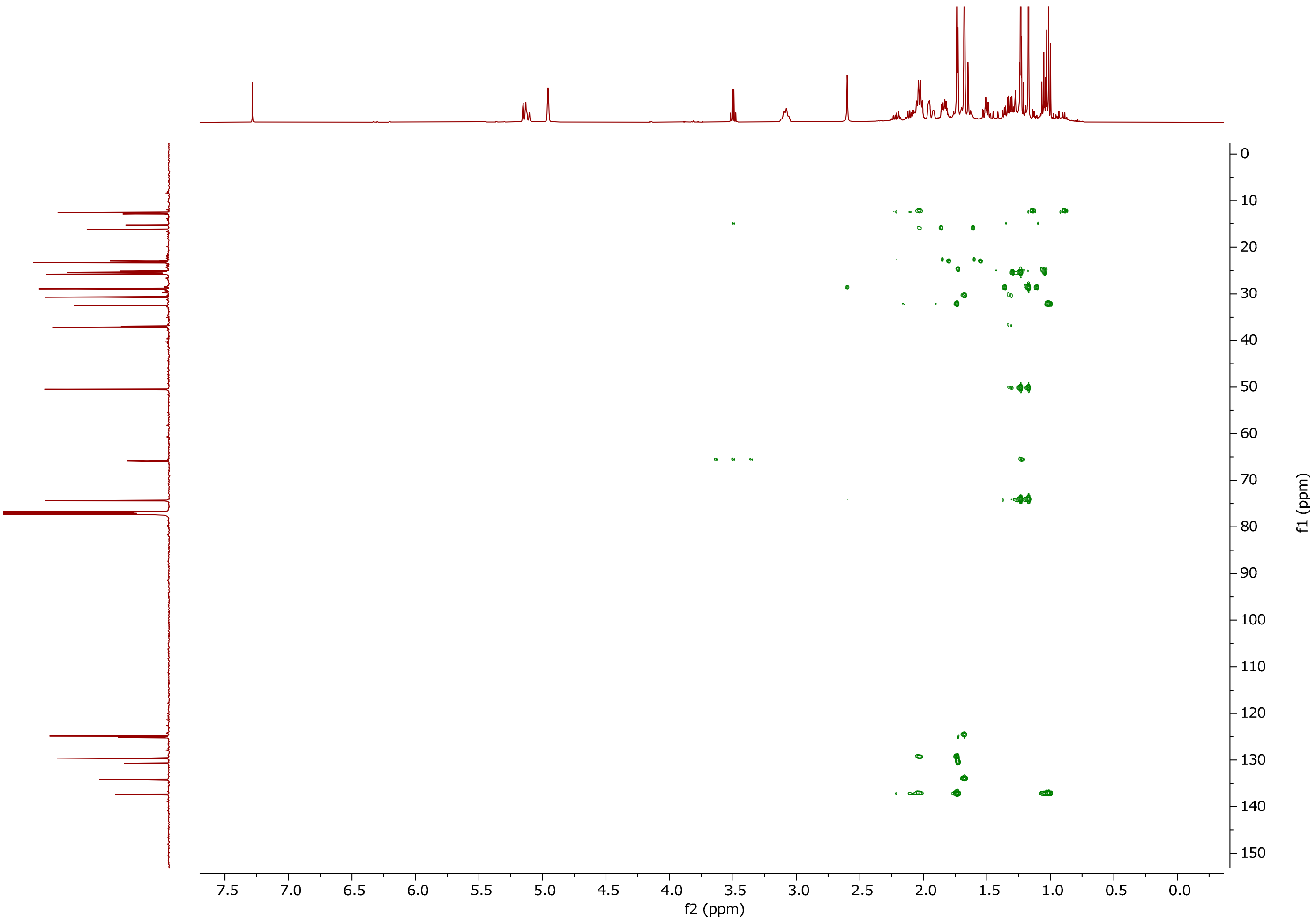

*Figure S9 ^{1}H-^{13}C HMBC spectrum of **42a** and **42b***

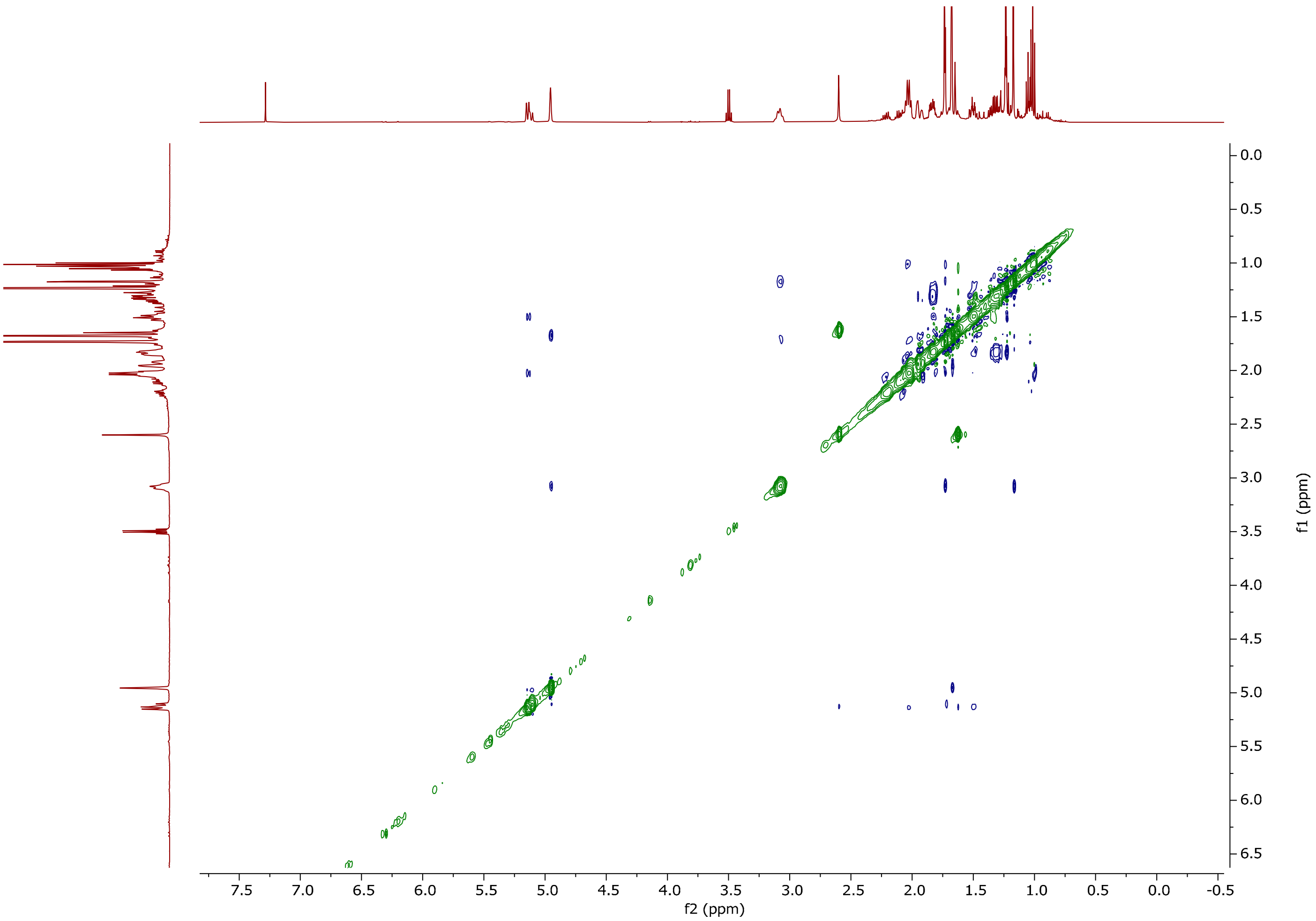

*Figure S10 ^{1}H-^{1}H NOESY spectrum of **42a** and **42b***
